# Inhalation of Nitrate of Silver

**Published:** 1858-07

**Authors:** W. H. Studly

**Affiliations:** Rochester, Ill.


ARTICLE VII.

### INHALATION OF NITRATE OF SILVER.

BY W. H. STUDLY, M. D., ROCHESTER, ILL.

I have noticed in several of the medical journals, from time to time, descriptions of more or less intricate instruments, contrived for the purpose of introducing nitrate of silver, in the powdered state, into the trachea and bronchial tubes. I have never seen any of these instruments, but I have thought them from the first, at least, unnecessary; inasmuch, as I have a far more simple, and I think fully as efficacious way, and at the same time unattended with the danger which I can fancy the instrument may be accompanied with. It strikes me that the grinding wheel of the caustic pulverizer may sometimes throw off a particle of considerable size from that very brittle pencil of nitrate of silver—of a size, at least, too large to enter the air passages of the lungs.

Then, again, it is not always desirable that the caustic should be introduced with full strength. Even Green, the great probanger, seldom uses a solution stronger than a drachm to the fluid ounce; and whenever caustic is used as an application to the other mucous surfaces, we are constantly varying the strength. Why is not the same liberty desirable in its application to the air passages?

My plan is this: I pulverize the nitrate of silver in a moderately heated wedgewood mortar, to an impalpable powder; I then triturate it with sugar of milk according to the strength which I desire—generally mixing them in the proportion of one part of the caustic to two of sugar of milk. This powder I put into a glass-stoppered jar of the pint or quart size, being careful to have the jar thoroughly dry by heating it. I place in the patient's mouth a glass or tin tube, one inch in diameter, and some eight or ten inches in length. Giving the jar a good shake and pulling out the stopper, I tell the patient to plunge the tube into the mouth of the jar and inhale. The cloud of powder which was seen floating in the jar, passes into and sprinkles the air passages thoroughly. From one to three inhalations at a time is sufficient, and about twice or thrice a week. The powder can be kept in good condition for about a month, the main trouble being the heating the jar every time you wish to use it, in order to drive off whatever atmospheric moisture may have collected. In all instances where it is desirable to go below the epiglottis with this remedy, I know of no way more efficacious than this, and being simple it is within the reach of all.

